# Gendered negotiations for research participation in community-based studies: implications for health research policy and practice

**DOI:** 10.1136/bmjgh-2017-000320

**Published:** 2017-06-07

**Authors:** Dorcas M Kamuya, Catherine, S Molyneux, Sally Theobald

**Affiliations:** 1 Health Systems and Research Ethics Department, KEMRI-Wellcome Trust Research Programme, Kilifi, Coast, Kenya; 2 The Ethox Centre, Nuffield Department of Population Health, University of Oxford, Oxford, Oxfordshire, UK; 3 Centre for Tropical Medicine, Nuffield Department of Medicine, University of Oxford, Oxford, Oxfordshire, UK; 4 Department of International Public Health Liverpool School of Tropical Medicine, Pembroke Place, Liverpool, UK; 5 Visiting Fellow Institute of Development Studies, University of Sussex, Brighton, UK

**Keywords:** Gender, power, ethics negotiations, constent process, developing countries

## Abstract

There is a growing literature documenting the complex realities of consent processes in the field, and the negotiations and ethical dilemmas involved. Much has also been written about how gender and power shape household decision-making processes. However, these bodies of literature have rarely been brought together to inform research theory and practice in low-income settings. In this paper, qualitative research (observation, focus group discussions and interviews) were used alongside large clinical community-based studies conducted on the Kenyan Coast to explore how gender and power relations within households and communities and between fieldworkers and communities shape consent processes and interactions. This exploration is embedded in relevant literature and the implications for community-based health research policy and practice are considered. Across diverse forms of households, we observed significant consultation on whether or not to participate in research. Although men are typically described as household decision-makers, in practice, decision-making processes are often far more nuanced, with many women using their agency to control, sometimes subtly, the decisions made. Where decisions are made without adequately consulting women, many find strategies to exercise their choice, in ways that safeguard important relationships within households in the longer term. We also found that the gender of field staff who typically conduct research activities in the field, including consent processes, can influence household dynamics and decision-making processes with important implications for the science and ethics of research. It is essential that frontline field staff and their supervisors are aware of the complex and gendered realities of consent processes at household level, and their implications, and that they develop appropriate context-informed approaches that support ethical practice.

**Key questions**

**What is already known about this topic?**

- Decision-making in many African communities is still highly patriarchal even though the gender equity gap is narrowing in.

**What are the new findings?**

- Applying a gender lens to consent processes helps us to understand how multiple layers of power interact with gender; and how power is exercised across different levels—within households and between participants and research staff.
- We demonstrate that fieldworkers' behaviours and roles shape ethics in practice, exhibiting simultaneous positions of institutional power and social vulnerability.

**Recommendations for policy**

- All research interactions are imbued with overt and subtle power relations, which a gender lens can help unpack.
- One approach to supporting frontline research staff is through reflective processes of building ethical mindfulness, an area that needs further theoretical and empirical investigation.
- A commitment to gender equity in employment of frontline research staff requires policies that address structural factors that disadvantage women and/or men.

## Introduction

### Consent and ethics

Considerable attention has been given to consent processes and research decision-making in low/middle-income countries, as well as empirical research documenting numerous challenges and approaches to address these. Suggested approaches include those aimed at increasing comprehension of research information, use of locally appropriate consent processes and responding to household decision-making dynamics.^1^ A foundational principle for consent is that autonomous individuals exercise their agency in making choices about research participation. An area largely unexplored in low/middle-income countries is how that agency is exercised for research consent within households, particularly in largely patriarchal and communitarian societies where multiple layers of authority and power exist.^2^ Although the importance of engaging household heads and community leaders at early stages of research has been highlighted in African settings,^3 4^ few studies have examined the effect of complex multilayered consent processes on the nature of social interactions between participants and frontline research staff. Understanding how research decisions are negotiated within diverse household structures, and with study teams, demands a critical examination of gender roles and relations.

### Gender and decision-making

A wealth of literature exists around gender and decision-making on many aspects of life, including in treatment-seeking behaviour, control and ownership of resources.^5–7^ The gender literature from low/middle-income country contexts suggests a shifting landscape in socially ascribed roles—including household decision-making—associated with narrowing gaps in gender inequity,^7–10^ increasing levels of education and economic independence for women,^11 12^ increasing democratic spaces for women and young people and increasingly equitable access, and control and use of resources at the micro-level and macro-level.^8 9 13^ However, there is great heterogeneity between and within communities, households and individuals, and hence considerable differences in the extent to which these positive effects are realised.^6 8 13^

Studies have shown that household decision-making patterns regarding research are complex negotiated processes influenced by a range of factors including: type and range of study benefits and compensations,^14 15^ the nature of study procedure(s) and risks and responsibility for study follow-ups.^16^ Furthermore, different individual household members can exercise their agency in different ways, including subtly as ‘silent refusals’ if their choices are not considered.^17 18^

### Role of fieldworkers in negotiating consent processes in the context of gender and power

A central group of research staff interacting with participants and household members are the frontline research staff, whose roles often include seeking consent and undertaking follow-up activities.^19^ Fieldworkers (FWs) employed in research institutions influence consent processes in important ways especially in populations with low literacy or limited exposure and experience with research. Being socially embedded in the communities hosting research, FWs bring critical awareness of socially acceptable behaviours and norms to study teams^20^ and can also influence the way the study is understood and taken up in communities.^21 22^ Some studies suggest that the gender of the FW may influence the nature of interactions, for example, the extent to which participants feel free to express themselves.^23^

### Vital gaps in the literature/knowledge base

Little attention has been given to the interplay between consent and gender in research decision-making. Moreover, although consent is described in the literature as a process rather than a one-off activity, there is relatively little information about post-consent negotiations.^24^ In this paper, we discuss findings of a social science study designed to explore the nature of interactions between FWs and research participants in community-based studies on the Kenyan Coast. During data collection, it became evident that significant negotiations were going on all the time across multiple levels, within households, between participants and study teams and within study teams, and that gender was a central theme in these negotiations. We examine how gender and power played out in negotiations, and consider the practical and theoretical implications for health research ethics in low/middle-income countries.

#### The study site

The study was conducted in a long-term large multidisciplinary research institute, the Kenya Medical Research Iinstitute-Wellcome Trust Collaborative Research Programme (KWTRP), with its headquarters on the Kenyan Coast. Research relevant to local, national and regional needs and priorities is undertaken with the 270 000 residents living n the area surrounding the county hospital, who also form the Kilifi Health and Demographic Surveillance System.^25^ A collaborative working arrangement with the county hospital management has led to long-term strategic support in health facilities, and research being integrated into the healthcare system. All studies conducted by the programme are approved by local and national scientific and ethics review committees. Community engagement activities are coordinated, managed and implemented with support from a dedicated group of community facilitators, the Community Liaison Group (CLG).

### Fieldworkers at the research centre

Nearly a third (n=334)* of the 800 staff are FWs, a cadre of staff with a minimum of 12 years of schooling, whose main roles include undertaking consent processes, follow-up activities and non-invasive study procedures (such as surveys, interviews, finger pricks, oral and nasal swabs). Most FWs are employed from the local community, a strategic decision as their roles include regular interaction with local residents and to provide employment to local community hosting research. FWs are regularly trained on their studies, as well as in communication skills and research ethics, including consent processes.

## Methods

The social science study conducted between February 2010 and March 2011 was designed to explore interactions across two very different community-based studies, which are described elsewhere.^18^ In brief, the studies were:

- Respiratory Syncytial Virus (RSV) study: An observational study involving entire households (n=47) examining RSV transmission patterns
- Malaria study: A malaria vaccine trial involving 900 children divided into two groups, 6–12 weeks old and 5–17 months old.

Thirty-six junior FWs and six senior FWs were employed across the two studies. All FWs came from and resided within the study population, and most were men (7/10 and 25/26 in the RSV and Malaria study, respectively). Their main roles included sharing initial study information with potential participants and carrying out follow-up activities. Different qualitative methods were used to capture voices, perspectives and realities of different players involved in the consent processes—participants, FWs, study coordinators and principal investigators (PIs).

Participant observation by DK of FWs doing their daily activities provided first-hand information on the context of FWs’ work and their interactions with households. The 19 FWs observed were selected from across studies to ensure diversity in activities and geographical areas.

Natural group discussions, where a discussion is held with naturally occurring groups such as household members^26^ were held with 16 adults (11 women and five men) from five purposively selected households (one female headed, one male headed and three extended households) participating in the RSV study, providing insights into the nature of interactions of adult members within a household. Women contributed minimally to these discussions. Consequently, for the Malaria study, focus group discussions with male and female respondents were held separately. Four Focus Group Discussions (FGDs) were held with 24 parents of participating children to maximise diversity in locality of residence, the FW allocated to them and age of child participating in the research.

Seven FGDs with FWs of varying seniority were held to explore views around the challenges that FWs faced in interactions with participants, and use of available support systems; and *s*even in-depth interviews were held with five study team members: the study PIs (n=2), coordinators (n=2) and a senior FW†.

### Data management and analysis

Data collection continued until a point of saturation where no new themes were emerging, and analysis was ongoing throughout the process. All cleaned transcripts were uploaded into Nvivo V.8.0, the software used to organise and manage the data. Data under each open code were grouped into descriptive themes, and codes were merged, deleted and created as more transcripts were added. Through this iterative process of analysis, further areas of enquiry were identified and incorporated into subsequent question guides. The descriptive codes were further grouped into broader analytical themes. Summary findings were presented to different cadres of staff in the case studies and to researchers at the centre. Gender emerged as a theme during data collection and preliminary analysis of initial data, and was added in subsequent research tools.

#### Ethics statement

This study was approved by the local and national institutional review boards (SCC No. 1463). Written informed consent was sought from all respondents in their preferred language. Only one person refused to be interviewed.

## Findings

Gender featured across three main levels of interactions and layers of negotiations: within households, between FWs and participants and within study teams. Underlying the nature of negotiations for research participation were household members’ hopes, fears and concerns about the studies (table 1), which fed into how men and women exercised their agency regarding research consent.

**Table 1:** Summary of participants’ hopes, fears and anxieties, by case study

Participants’ views	RSV study	Malaria study
**Participants’ hopes**		
High quality healthcare	Free healthcare for participants and family members during trial; provision of community level benefits (eg, boosting health facilities).	As with RSV study.
Research optimism, that is, the research will be successful	Positive research results will lead to a vaccine being available soon. Contributing to benefits for future generations.	Vaccine perceived as already working. Pleased to be pioneer beneficiary of a ‘successful vaccine’. Will benefit future generations.
**Fears and anxieties**		
Study procedures	NFS safety. Concern about transmitting infections, frequency and depth of NFS could lead to brain damage and future health problems.	Worries of severe adverse events and volumes of blood. Fears that infantometer for weighing babies symbolises a coffin.
Association with KEMRI activities	Worries that the research programme isinvolved in devil worship, linked to wealth, free study benefits.	Same as RSV study, worries also associated with blood samples.
Particular sensitivities/confusions in ICF information	Terminologies and concepts in consent forms, for example, information about RSV.	Anxieties around Information and Consent Forms (ICF) information, for example, randomisation, trial, placebo, compensation and confidentiality.
FW competence	FWs’ ability to perform NFS safely.	

FW, fieldworker; NFS, nasopharyngeal flocked swab; RSV, respiratory syncytial virus.

### First steps: engaging household heads; an important step in the community receiving a study

The PIs anticipated there would be initial challenges with acceptability of both studies. The RSV study PIs were particularly concerned that they only had a very narrow window of a 5-month RSV epidemic season. Household heads were engaged through village and household level meetings in all six participating villages with support from the CLG and study FWs. This was important in recognising the critical role that household heads played in accepting or rejecting the study, and in addressing concerns, fears and rumours circulating in the community about the study.

#### Within household negotiations

In this largely patrilineal community, male household members are generally expected to make most decisions for the family, including treatment-seeking decisions, control, access to and use of household resources. This pattern includes research decisions:

‘ …usually in our culture a woman is married into the man’s home, she can’t decide anything because she is a visitor …So (in terms of) responsibilities, the man is the head of everything…” (FW1_male_CSB/FGD10)

Research participation was perceived to involve commitment and responsibility for follow-up schedules and for handling any side effects. People regularly expressed fears and reservations in research where numerous potential side effects are noted in consent forms (the Malaria study), or the study procedures were unfamiliar, uncomfortable or disliked (taking of nasal-pharyngeal flocked swab (NFS) in the RSV study).^21^ The range and depth of negotiations and decision-making patterns appeared to be influenced by household arrangements, in particular whether households were nuclear or extended and male or female headed.

##### Extended households

Most households on the Kenyan Coast are extended households,^27^ with several generations living together, and where the eldest male member is generally considered the household head and main decision-maker. Consent processes therefore began with the male household head (HHH).

…When you get a household head whom when you explain they understand well, it used to be very easy for them to help you in explaining to the others; so that made our work easier (FW4_male_CSA/FGD06)

Household members supported involving senior male members in research decisions because of decision-making norms and concerns about the research and who would take responsibility should side effects be observed.

‘…and the mother may say, ‘I have agreed,…but my husband is not present, he must know and decide; I am ready, and even if I don’t want (the study) and my husband wants what say do I have?’” (Pax3_female_CSB/FGD13).

In this community, blame towards the male household members is often less severe than that directed at women.^16^ A man making a decision about a child’s research participation was perceived as underscoring his biological paternity and ownership of the child. On the other hand, there could be severe negative consequences for women failing to involve or disobeying their husbands’ decisions, including being ‘chased away’ from home.^13^

‘… as the mother you will be informed (about the study) and you will think about it…if I go alone my husband will come and quarrel with me’. So I will wait for my husband to come and inform him so he understands’ (Pax5_female_CSB/FGD14)

##### Nuclear families

Decision-making processes for male-headed nuclear families mirrored that of the extended household, with the male household generally making the final decision. Where the male household head was deceased, the mature eldest son in the family (especially if married) was considered the legitimate household (HH) head even if his mother was alive. Where there was no son or the eldest son was too young, a clan-delegated household head would usually make decisions in consultation with the first wife of the deceased. Wider cycles of consultation were usually reported in female-headed households, often involving children (especially the eldest son), siblings (especially brothers) and grandparents.

…So we wanted everyone to give their own views. So the infant’s mum said ‘I am not ready, I cannot decide on my own, there is my child, I will inform him because he is the one who is learned [ie educated] whom I depend on… she openly said that her son refused and explained to us that ‘I told him that I cannot undermine him, so I also withdrew’ (FW1_male_CSA/FGD06)

Across all household types, extensive consultations within households and with significant others were reported. Where there were differences, women would often defer to the man’s choice/decision. However, as we have reported elsewhere, other patterns of negotiations and influences also emerged including ‘silent refusals’ in which participants regularly avoided follow-up activities/procedures, often hiding their refusal with plausible and understandable issues and delays.^18^ As noted in a previous study on treatment seeking for seriously ill children, pronounced roles for elder women and grandmothers were identified,^13^ particularly for female-headed households.

…he [fieldworker] gave me time to think about it saying ‘take your time and think about it’. I told him to wait until my child’s grandmother comes so that I ask her about it too and see if she will agree to join…and when the grandmother came, I told her and she said its fine, ‘you can enrol’. I came and told him, and he said it’s fine I can join. (Pax1_Female_CSA/HH2)

#### Fieldworkers’ and participants’ interactions

Most FWs in the two community-based studies were men, 7/10 and 25/26 in the RSV study and Malaria study, respectively. Most household interactions were therefore between male FWs and participants. One reason for fewer female FWs is that fewer girls than boys attain 12 years of schooling in this region and fewer still attain the mean grade of C, the main qualification criteria for a FW position at the institution. In addition, community-based FW job requirements include riding motorbikes/bicycles and working late hours, which could discourage some potential female applicants. PIs in both studies described taking extra steps to try to recruit qualified female FWs, including publicly encouraging female applicants, assigning households which were near their homes and providing car transportation for faraway household follow-ups.

### Fieldworkers too familiar, not taken seriously

Most of the FWs were already known to households because they were recruited from and resided within their communities.^21^ Familiarity and informality facilitated discussions about specific studies but also led to blurred boundaries between social and professional conduct.

…sometimes you know that person who is too familiar with you, you may tell them something and treat it causally… [she/he] may forget that professional part of you or that work you do. (FW3_male_CSA/FGD06)

Disrespect was discerned in the way some participants and FWs talked to each other, and in casual interactions imbued with cajoling and sometimes sexual undertones. (Male) FWs expressed vulnerability in situations where they were being ‘flirted with’ because there was potential for loss of trust in the FWs, which could contribute to damaged reputation for the FW and the institution.

…It had reached a level where I felt defeated. There was one day I went to collect samples, so I wanted to take the temperature, so that girl… opened all the buttons up to here [navel]… I told her ‘ahh my sister, do you have to open all the buttons to take a temperature measurement?’…I did not feel good… I think it was one way of chasing me from the household (FW1_male_CSA/FGD06)

When faced with these situations, some FWs reported discussing it with their seniors or seeking advice from fellow FWs. Others simply carried on and waited for the end of the study in the hope that it would not blow up, and others informed household members in an effort to re-establish trust and respect. Working in participants’ homes presented a particular level of vulnerability for FWs in this regard, since they had to be welcomed into those homes and support was essential if follow-up activities—and the study—were to continue. One FW narrated an incident where his repeated late-hour visits to a household of an estranged couple led to him almost being beaten up by the husband, who assumed that the FW was using study interactions as a strategy to develop close relationships with his wife. Additionally, some of the FW’s research-related roles overlapped with social roles ascribed to male household heads, such as providing fare reimbursements and access to healthcare. This might have contributed to simmering jealousies from married men towards male FWs, who were regarded with their full time employment as having relatively high status in the community. Male FWs felt frustrated with having to repeatedly explain their roles, but recognised that it came with being socially embedded in the community.

‘…so, anytime a mum comes to you, and asks for help, she can request even for advice, you have to think, what should I do, because you don’t want to cause problems with her husband…,” (FW_male_CSB/IDI07)

Handling different individuals across different households required FWs to be aware of appropriate responses to household dynamics, and sensitive to issues that matter to household members. As one FW said "we had to know these households, I tell you, what worked in one household did not always work in another, each day was a learning lesson…,” (FW1_male_RSV-study/FGD05).

## Discussion

We observed over the course of this study the way gender and power relations shaped ongoing consent processes in two different community-based studies on the Kenyan Coast. There were complex negotiations within and across different household types, and between different generations and multiple actors, including participants, other household members and FWs. Our analysis synthesised four key themes from the findings, which are discussed next in light of relevant literature: blurred boundaries in negotiations; examining gendered consent processes as an approach to unpack relational ethics; vulnerability of FWs in interactions with participants; and the messy realities of FW’s complex and ethically imbued roles.

### Blurred boundaries

The patterns of negotiations show that consent processes and decisions regarding research participation over the course of the research were changing all the time, and shaped by the nature of the negotiations. Within the Kenyan Coastal community we were working in, there is in fact a wide range of household types that are hard to classify into discrete typologies. Each structure is shaped by gender and generational norms which influence negotiations. Across all household types and generations, household heads are recognised as key players shaping decisions taken by others, even where their sphere of influence may be limited. This type of layering of decision-making for research has been documented in other similar settings,^24 28^ and illuminates the complex gendered nature of consent processes.

#### Gendered consent processes: importance of relational ethics

As our findings show, the patterns of negotiations varied widely within and across household arrangements, although there was a pronounced role for male household members. This dominant role could be because research is relatively unfamiliar, people are wary of bearing responsibility for research-related risks and there is deep respect for elders. Thus, household head’s permission for research teams to visit their homes is important. Nevertheless, consultation about consent was common across household types, and while male member’s choices were privileged, women (and youth) often found ways to act on their preferences, for example, avoiding some of the study procedures they were uncomfortable with as we have documented elsewhere.^18^ Thus, applying a gender lens to consent processes helps us to understand the way in which social norms around gender shape negotiations for research. While consent guidelines show high regard for individual autonomy in consent processes, in reality, autonomy is enacted within an ever-shifting balance of decision-making power at household levels. Universal codes, principles and regulations that focus on the individual and that do not explicitly address relational ethics need to be applied sensitively and appropriately across contexts^29^ such as coastal Kenya where community and kinship ties are strong.

#### Power relations in participant-fieldworker relations

FWs entering and conducting study activities in participants’ homes had power due to knowledge, access to resources and the respect and status they enjoyed. However, they were also in positions of vulnerability: by working in participants’ homes and carrying out study procedures, FWs were placed in social spaces of intimacy. Here, participants had subtle power to determine whether FWs would be welcomed, and by extension whether the study would be successful. That the gender of FWs interacting with participants’ matter in the conduct of research has been reported for qualitative interviews,^30^ in participant recruitment and follow-up,^31^ as essential in respecting cultural norms.^32^ The conduct of male FWs in households was scrutinised and any indication of ‘misconduct’, including being overly friendly, was likely to influence how they were viewed in the households and in the community. Some study procedures (such as taking of temperature and of NFS) required FWs to breach local norms, with many male FWs reported having to seek permission from household authorities before carrying out such procedures to avoid misinterpretation. These factors contributed to male FWs feeling anxious in situations they would ideally have institutional authoritative power over participants. Vulnerability of female FWs was less pronounced. This may have been due to low number of female FWs employed in our community-based studies. The research centre’s approach to employing female FWs could unintentionally deny female FWs opportunities to work in community-based studies. A commitment to fairness in employment of FWs would require policies that address structural factors that disadvantage female or males; for example, revising minimum qualifications for certain types of jobs, instituting gender-based quota systems of employment, providing flexible working hours for parents, although some of these strategies could be expensive or complex to implement.

#### FWs are doing ethics in the field: the need for critical reflection

The unique position of FWs in research implementation means they had multiple roles and interests: as gatekeepers of the community they come from and work within; of cultural brokerage for their employers; and their own interests to advance their careers and maintain their status in the community. How they mediate between these multiple roles, power positions and interests is important for culturally sensitive and ethically sound research conduct.^32^ Thus, FWs were doing moral work, weighing up what would be the right thing to do in each situation they encountered. Our findings illustrate this weighing up presented several dilemmas where social norms and research guidelines were in tension, such as whether to respect a household head’s decision while other members’ choices differed, and particularly given the cultural norm of respect towards elders. FWs at the interface are therefore always ‘brokering across cultures’, mediating between research and community norms in order to minimise conflict,^32^ but do so with limited power themselves over research resources and decision-making. A critical consciousness of the morally imbued roles of FWs is needed, in both the FWs (to develop a critical and analytical sense of how their actions might affect others and implementation of the research) and those who supervise and support them (to understand the complex ethical dilemmas which frontline research staff face and how best they can be supported). Several approaches to supporting frontline research staff have been suggested.^18 23^ A reflective process that builds ethical mindfulness^33^ for frontline research staff could be built into fieldworker support processes; however, further theoretical and empirical investigation of how this might be done its usefulness and effectiveness is needed.

#### Study limitations

Few female FWs were employed in the two community-based studies and hence it was difficult to explore how female FWs' interactions were shaped by gender and power. The initial intention of the study was to explore ethical issues for frontline research staff; gender emerged as one of the key themes rather than being the starting point of enquiry, and other factors that may shape gender negotiations—such as education, resource control and income levels—could have been further explored.

## Conclusion

Frontline staff face multiple ongoing challenges in seeking research consent for individuals and whole household. They work with diverse range of household types and often encounter ever-shifting decision-making processes which are shaped by gender and generations of those involved in research. They are thus essentially always negotiating about research and their own positions in interactions with individuals, households and communities. The gender of the FW and evolving relationships with participants add another layer of complexity, sometimes highlighting authority and power that FWs assume in their roles, and at other times rendering them vulnerable. Thus, FWs’ conduct and interactions are shaped by gender and power relations, and are critical to both appropriate consent processes and robust science. It is important to develop supportive strategies that build FWs' abilities to reflect on their positionality and how it influences others. This can support fieldworkers to make appropriate choices in the context of complex and changing social situations, which is essential to ensuring ethical practice in everyday research encounters.

## Footnotes

* As of March 2011.

† Study had 1 senior FW.
